# RNA m^6^A modification, signals for degradation or stabilisation?

**DOI:** 10.1042/BST20230574

**Published:** 2024-04-17

**Authors:** Guifeng Wei

**Affiliations:** Department of Biochemistry, University of Oxford, Oxford OX1 3QU, U.K.

**Keywords:** IGF2BP, METTL3, mRNA stability, *N*^6^-methyladenosine, YTHDF

## Abstract

The RNA modification *N*^6^-methyladenosine (m^6^A) is conserved across eukaryotes, and profoundly influences RNA metabolism, including regulating RNA stability. METTL3 and METTL14, together with several accessory components, form a ‘writer’ complex catalysing m^6^A modification. Conversely, FTO and ALKBH5 function as demethylases, rendering m^6^A dynamic. Key to understanding the functional significance of m^6^A is its ‘reader' proteins, exemplified by YTH-domain-containing proteins (YTHDFs) canonical reader and insulin-like growth factor 2 mRNA-binding proteins (IGF2BPs) non-canonical reader. These proteins play a crucial role in determining RNA stability: YTHDFs mainly promote mRNA degradation through different cytoplasmic pathways, whereas IGF2BPs function to maintain mRNA stability. Additionally, YTHDC1 functions within the nucleus to degrade or protect certain m^6^A-containing RNAs, and other non-canonical readers also contribute to RNA stability regulation. Notably, m^6^A regulates retrotransposon LINE1 RNA stability and/or transcription via multiple mechanisms. However, conflicting observations underscore the complexities underlying m^6^A's regulation of RNA stability depending upon the RNA sequence/structure context, developmental stage, and/or cellular environment. Understanding the interplay between m^6^A and other RNA regulatory elements is pivotal in deciphering the multifaceted roles m^6^A plays in RNA stability regulation and broader cellular biology.

## Introduction

*N*^6^-methyladenosine (m^6^A) was originally identified and partially characterised in the 1970s [1–3]. Since then, it has emerged as one of the most prevalent internal RNA modifications. Notably, m^6^A has been found in a wide range of organisms, spanning mammals, fish, insects, plants, and yeast. Furthermore, this modification is observed in nearly all types of RNA, encompassing mRNA, long noncoding (lnc)RNA, enhancer (e)RNA, promoter upstream transcript (PROMPT), repeat RNA, circular RNA, microRNA, rRNA, snRNA, and viral RNA.

**Figure 1.**
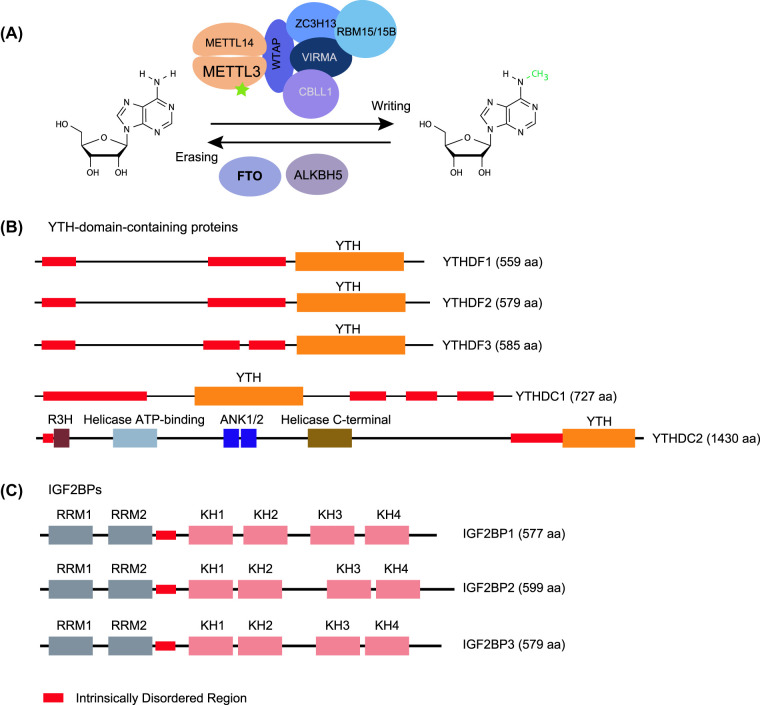
m^6^A writers, readers, and erasers. (**A**) The RNA modification m^6^A is deposited by a methyltransferase complex comprising a heterodimeric core consisting of METTL3 and METTL14, along with additional accessory subunits including WTAP, VIRMA, ZC3H13, CBLL1, and RBM15/15B. METTL3 functions as the only catalytic subunit, converting adenosine (A) to N^6^-methyladenosine (m^6^A). Below, two m^6^A erasers, FTO and ALKBH5, are illustrated. (**B**) The domain architecture of canonical m^6^A readers, YTH-domain-containing proteins (YTHDF1/2/3 and YTHDC1/2) which directly recognise m^6^A through their YTH domain. The protein sizes shown on the right are in accordance with annotations in the human genome. Note: recent evidence suggests that YTHDC2 may bind to RNA independently of m^6^A [82], contrasting with the original finding [83]. (**C**) The domain architecture of non-canonical m^6^A readers IGF2BP proteins (IGF2BP1/2/3) which recognise m^6^A through their KH domain. The protein sizes shown on the right are in accordance with annotations in the human genome.

METTL3, the enzyme responsible for catalysing m^6^A deposition, was identified in the mid-1990s [4]. Throughout the 2010s, several other proteins were found to form a complex with METTL3, acting together to facilitate the deposition of m^6^A onto RNA. This complex is now referred to as the m^6^A ‘writer' complex and consists of a heterodimeric enzymatic core, comprising METTL3 and METTL14, along with accessory subunits including WTAP, CBLL1/HAKAI, ZC3H13, VIRMA, and RBM15/15B (Figure 1A) (reviewed in [5]). m^6^A is particularly enriched near the stop-codon and internal long-exon regions of mRNAs in many species [6,7]. Structural studies revealed that within the ‘writer' complex, METTL3 serves as the sole catalytic subunit, whereas METTL14 possesses a degenerate active site, playing an essential role in preserving the structural integrity of the complex and facilitating substrate recognition [8–10]. Although in recent years other m^6^A-modifying enzymes, including METTL16, METTL5, and ZCCHC4, have been identified, the vast majority of m^6^A on mRNA and lncRNAs is METTL3 dependent [11,12] and hence form the primary focus of this review.

In 2011, the enzyme FTO was found to have the ability to demethylate m^6^A, indicating that this modification is dynamic [13]. Subsequently, a second m^6^A demethylase ALKBH5 was identified through homology search and functional characterisation [14]. These two proteins are now referred to as m^6^A ‘erasers' (Figure 1A).

Proteins directly recognising m^6^A are typically identified using synthetic m^6^A-containing RNA as a bait. RNA pull-down and subsequent mass spectrometry analysis are applied to isolate and identify the interacting proteins [6,15,16]. To date, two classes of proteins, collectively referred to as m^6^A ‘readers', have been identified using this method: canonical readers (YTH-domain-containing proteins: YTHDF1/2/3 and YTHDC1/2) (Figure 1B) and non-canonical readers. These non-canonical readers include insulin-like growth factor 2 mRNA-binding proteins (IGF2BPs) (IGF2BP1/2/3) (Figure 1C), along with numerous other RBPs identified in RNA pull-down analysis such as FMR1 [16,17], RBM45 [18], PRRC2A/B [19,20], and TDP-43 [21]. These proteins play crucial roles in conveying the functional impact of m^6^A modifications by interfacing with various downstream molecular pathways.

The deposition of m^6^A onto RNA is widely considered to be co-transcriptional, supported by multiple lines of evidence based on sequencing, imaging, and biochemical data [22–28]. m^6^A continues to play a multifaceted role in nearly all aspects of RNA metabolism throughout its life cycle, including splicing, stability, export, structure, and translation. Indeed, one of the most well-documented functions of m^6^A is its role in facilitating RNA degradation. However, there are many instances where m^6^A is also implicated in maintaining RNA stability, a phenomenon observed in various developmental and disease contexts. This review will specifically focus on the differing roles of m^6^A in the regulation of stability in different types of RNA, with the broader spectrum of m^6^A functions extensively reviewed elsewhere (reviewed in [12,29–32]).

## m^6^A promotes RNA degradation

### m^6^A promotes RNA degradation in the cytosol by YTHDFs

**Figure 2.**
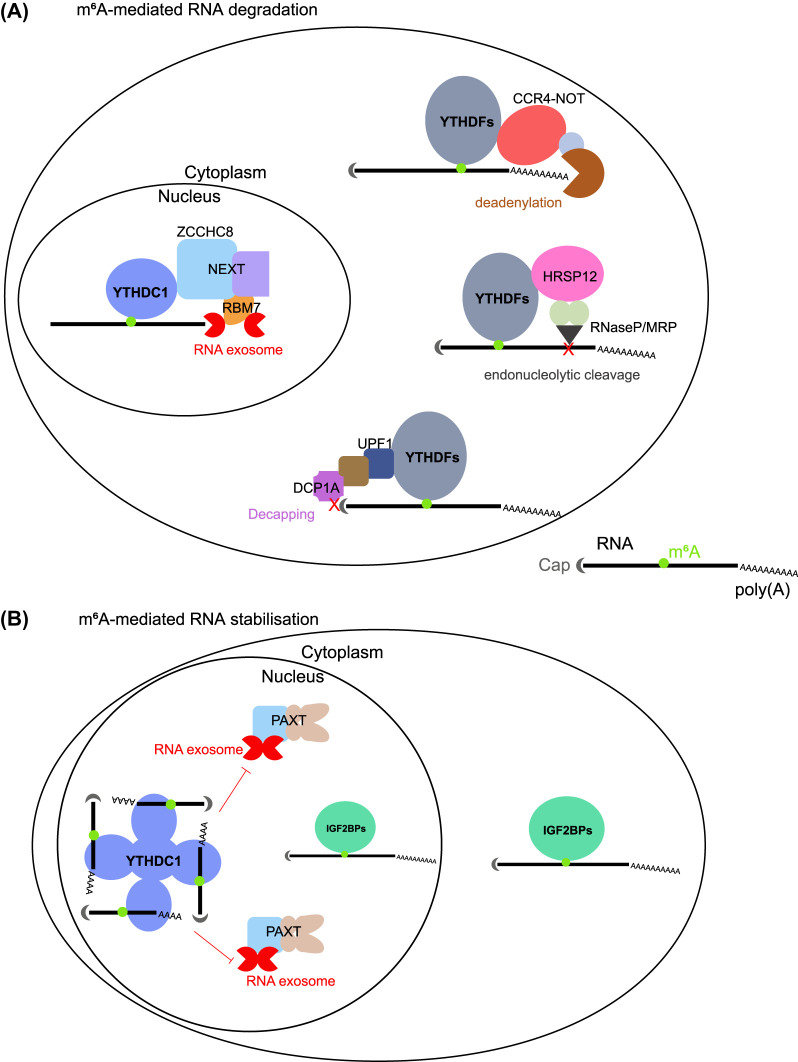
Regulatory pathways linked to m^6^A-mediated RNA stability regulation. (**A**) m^6^A-mediated RNA degradation: In the nucleus, YTHDC1 interacts with the nuclear exome targeting complex (NEXT) to facilitate the degradation of chromatin-associated regulatory (car) RNAs. In the cytoplasm, YTHDF proteins (YTHDF1/2/3) interact with three distinct pathways for RNA degradation, including the CCR4–NOT deadenylation complex, HRSP12–RNase P/MRP endonucleolytic cleavage pathway, and UPF1–DCP1A RNA decapping enzymes. Note: All YTHDFs are confirmed to interact with the CCR4–NOT complex; YTHDF2, YTHDF1/3 marginally, associate with RNase P/MRP pathway; YTHDF2 also engages with UPF1 pathway. (**B**) m^6^A-mediated RNA stabilisation: In the nucleus, YTHDC1 binds to m^6^A-containing mRNAs and forms nuclear condensates, thus protecting RNA from poly(A) tail exosome targeting complex (PAXT)-mediated decay. In the cytoplasm, IGF2BPs (IGF2BP1/2/3) binds to m^6^A-containing mRNAs to enhance RNA stability. Note: IGF2BPs could also function within the nucleus to enhance RNA stability.

The well-documented function of m^6^A is its role in promoting RNA degradation, which is predominantly facilitated by m^6^A ‘reader’ proteins that bridge with downstream molecular pathways. In the cytosol, the YTHDF family of proteins, particularly YTHDF2, preferentially recognise m^6^A-containing RNAs and subsequently direct them towards degradation pathways [33]. YTHDF2 has been discovered to engage with at least three prominent pathways to facilitate the degradation of m^6^A-containing mRNAs (Figure 2A).

Firstly, through co-immunoprecipitation (co-IP) and mass spectrometry assays, YTHDF2 was found to interact with the CCR4–NOT deadenylation complex, marking the first described molecular pathway linking m^6^A-containing mRNAs to degradation [34]. Secondly, a more novel and recent study uncovered that m^6^A-containing RNAs which possess HRSP12-binding sites in close proximity to RNase P/MRP-directed cleavage sites are preferentially targeted for endoribonucleolytic cleavage through the YTHDF2–HRSP12–RNase P/MRP axis [35]. Notably, this axis is also responsible for the degradation of a subset of m^6^A-containing circular RNAs that lack a poly(A) tail. A recent third body of work demonstrated that YTHDF2 also interfaces with UPF1, an RNA helicase renowned for its involvement in nonsense-mediated decay (NMD). However, the YTHDF2–UPF1 axis is likely to operate by recruiting the RNA decapping enzyme DCP1A [36], rather than via NMD. The latter two mechanisms require further validation across various contexts.

The presence of multiple cellular pathways dedicated to m^6^A-mediated RNA degradation may be attributed to the abundance and diverse RNA species and/or sequence contexts, ensuring their efficient clearance. It is worth noting that recent studies have proposed a redundancy in the function of m^6^A reader proteins YTHDF1, YTHDF2, and YTHDF3. This redundancy is likely attributed to their striking similarities in protein sequence, domain architecture, interacting partners, and the RNAs they bind to [37–39]. Redundant or overlapping functions of YTHDF proteins are supported by observations *in vivo* (during mouse gametogenesis and postnatal viability) and *in vitro* (mouse embryonic stem cells (mESCs)) [40]. However, a recent study found dynamic levels of *O*-GlcNAc modifications appearing on YTHDF1/3 proteins, but not YTHDF2, dependent on background, indicating that they do have diverse functions [41]. Nevertheless, it is important to interpret RNA stability data from triple YTHDF1/2/3 knockdown/knockout experiments with care, as depletion of all three YTHDF proteins results in an increase in cellular P-body formation in HeLa cells, consequently leading to the global stabilisation of most mRNAs which is not strictly dependent on m^6^A [42].

YTHDF proteins have also been found to recognise m^6^A within viral RNA and play regulatory roles during viral life cycles [43–46]. However, the specific impact of YTHDFs on viral RNA stability, akin to their role in host cellular RNAs, remains controversial. For instance, Tsai et al. [43] reported that YTHDF2 binding to m^6^A sites on HIV-1 RNA significantly stabilises these viral RNAs, boosting viral infection, but conflicting studies have suggested inhibitory roles of YTHDFs in HIV-1 infection [44,45]. These findings underscore the need for further studies to elucidate the precise context-dependent mechanisms underlying the functions of each YTHDF protein.

### m^6^A promotes RNA degradation in the nucleus by YTHDC1

In the nucleus, the YTH-domain-containing protein YTHDC1 stands out as one of the best characterised canonical m^6^A readers [47]. Co-IP experiments with YTHDC1 have found it associated with splicing regulators [27], as well as ZCCHC8 [48,49], a core component of the nuclear exosome targeting complex (NEXT). NEXT plays a critical role in the degradation of non-polyadenylated chromatin-associated regulatory (car) RNAs, including eRNAs and PROMPTs. In turn, ZCCHC8 has been reported to reciprocally interact with YTHDC1 in experiments employing stable isotope labelling in cell culture (SILAC) and mass spectrometry [50]. Furthermore, various studies employing distinct methodologies have identified the core components of NEXT, RBM7, or ZCCHC8, as proteins linked to m^6^A [51]. The YTHDC1–RNA exosome axis has also been implicated in the degradation of many nuclear m^6^A-containing RNAs (Figure 2A), including eRNAs and PROMPTs in mESCs [49], immunoglobulin heavy chain locus-associated lncRNA (SμGLT) in B cells [52], and *C9ORF72* repeat RNA in ALS/FTD patient-derived induced pluripotent stem cell (iPSC)-differentiated neurons [53]. On the contrary, Lee et al. [54] found that YTHDC1 depletion did not alter eRNA stability in human cell lines (MCF7, K562, and HeLa). Instead, they found that m^6^A-eRNA recruits YTHDC1 to enhancer regions to stimulate enhancer activity and gene transcription by facilitating transcriptional condensate formation. The discrepancy between these studies may arise from differences in cell types (pluripotent vs differentiated), the technical methodologies (chronic knockout vs acute depletion) used, and the lack of m^6^A stoichiometry measurement on these eRNAs studied.

## m^6^A enhances RNA stability

### m^6^A protects RNA from degradation by IGF2BPs

Another category of m^6^A-interacting proteins is the insulin-like growth factor 2 mRNA-binding proteins (IGF2BPs; including IGF2BP1/2/3). These were initially identified through the use of synthetic single-stranded m^6^A-containing RNA (GGm^6^ACU) as a bait for RNA pull-down, followed by mass spectrometry analysis [15]. Unlike the YTH domain, the K homology (KH) domains of IGF2BPs are responsible for m^6^A recognition (Figure 1C). A recent structural study has further revealed that the hydrophobic groove within KH4 of IGF2BPs contains a dedicated and evolutionarily conserved structural element that conveys m^6^A specificity. While this m^6^A recognition is independent of the underlying RNA sequence context, there is a preference for GGAC, with double mutations of V523I/P524S shown to significantly reduce cognate binding [55]. Interaction between IGF2BPs and m^6^A is observed only at certain m^6^A sites, as revealed by cross-linking and immunoprecipitation followed by high-throughput sequencing (CLIP-seq) analysis [15,55], in contrast with YTH-domain-containing proteins. This suggests that m^6^A does not represent a universal layer of regulation in IGF2BPs target selection. Additionally, it is important to note that the binding affinity of IGF2BPs for m^6^A-containing RNAs is considerably weaker compared with that of YTH domains [47,55]. Moreover, RNA structural data suggest that IGF2BPs may recognise the structural changes induced by the so-called ‘m^6^A-switch [56]'. In this scenario, m^6^A alters local RNA structures, thereby facilitating the binding of RNA-binding proteins such as hnRNPC [57] and hnRNP A2B1 [58]. Further experiments are needed to elucidate this aspect regarding the precise mechanisms through which IGF2BPs execute RNA stabilisation.

In contrast with the destabilising role of the YTHDF–m^6^A axis, IGF2BPs play a role in promoting the stability of their target RNAs in an m^6^A-dependent manner (Figure 2B). This axis was determined by experiments where knockdown/knockout m^6^A writer complex led to reduced stability of m^6^A-containing RNAs, or knockdown/knockout m^6^A eraser proteins resulted in increased stability of certain m^6^A-containing RNAs, with further biochemical experiments functionally confirmed the stabilising role of IGF2BPs–m^6^A across multiple cellular, physiological, and pathological contexts, including human cancer cells (hepatocellular carcinoma: HepG2, cervical cancer: HeLa, prostate cancer: 22Rv1, acute myeloid leukaemia) [15,59], as well as mouse oocytes, during early embryonic development [60], and postnatal liver development [61].

Interestingly, while m^6^A plays a stabilising role for zygotic degraded m^6^A-containing maternal transcripts before fertilisation, these transcripts with m^6^A are degraded more quickly after zygotic genome activation than those without m^6^A. These opposing effects of m^6^A on maternal transcripts during the maternal-to-zygotic transition suggest the involvement of distinct readers or pathways [60,62–64].

### YTHDC1 protects m^6^A-containing RNA from degradation

YTHDC1 has also been implicated in stabilising m^6^A-containing RNAs. For instance, in acute myeloid leukaemia, YTHDC1 binding of nuclear m^6^A transcripts enables the formation of nuclear condensates that protect nuclear mRNAs such as *MYC* from poly(A) tail exosome targeting complex (PAXT)-mediated degradation (Figure 2B) [65]. Additionally, during mouse preimplantation development, YTHDC1 binds to a small group of m^6^A-containing genes, sustaining their RNA stability [66]. However, none of these studies demonstrated the effect of YTHDC1 on RNA stability regulation depends on the quantity of m^6^A sites or m^6^A levels present on these RNAs. Furthermore, these studies raised another question regarding the extent to which YTHDC1 binds m^6^A-containing transcripts for either enhanced stability or destruction in different cellular contexts. Specifically, for nuclear m^6^A-bearing transcripts, how does YTHDC1 distinguish between those targeted for degradation and those marked for protection?

### m^6^A in the poly(A) tail of VSG RNA is required for its stability in *Trypanosoma brucei*

In the parasitic kinetoplastid *Trypanosoma brucei*, remarkably approximately half of the m^6^A modifications in VSG RNA is situated within its poly(A) tail. These m^6^A modifications are then systematically eliminated from the VSG poly(A) tail prior to RNA deadenylation and degradation [67]. Intriguingly, a 16-mer *cis*-acting motif positioned in the 3′-UTR of VSG plays a crucial role in the deposition of the poly(A) tail m^6^A, with excision of the motif resulting in lack of m^6^A, and the subsequent acceleration in the rate of deadenylation and degradation [67]. Given that trypanosomes lack an orthologue of METTL3 and m^6^A is found in the poly(A) tail instead of the canonical DRACH motif [67], this is highly suggestive of the presence of an alternative m^6^A writing mechanism and possibly different reading mechanisms within this species.

## RNA stability regulated by other non-canonical m^6^A readers

The proline-rich coiled-coil 2 (PRRC2) family protein PRRC2A has been identified as a neuronal cell-specific m^6^A reader protein exhibiting a preference for m^6^A [19]. PRRC2A stabilises Olig2 mRNAs by binding to the m^6^A within the coding sequence region of Olig2 and this stabilisation effect is reversible through the action of the m^6^A demethylase FTO. However, another recent study showed that PRRC2A recognises spermatogonia-specific transcripts and down-regulate their RNA abundance [68], thereby maintaining the spermatocyte expression pattern during the meiosis prophase. It is important to note that this analysis was conducted on a transcriptome-wide scale rather than focusing on individual transcripts. The divergent roles observed for PRRC2A in different tissues underscore the tissue-specific nature of its function. Recently, another PRRC2 family protein PRRC2B has also been identified as an m^6^A reader and stabilises Sox2 mRNA in an m^6^A-dependent manner [20].

In addition, work in *Drosophila* has identified FMR1 as an m^6^A reader through a short m^6^A-modified RNA oligo-based approach [69]. Beyond its documented role in translational regulation, FMR1 is implicated in maternal RNA decay during *Drosophila* embryogenesis [69], mediated at least in part by m^6^A. In the mouse cerebral cortex, FMR1 regulates the stability of its m^6^A-marked mRNA targets through binding to YTHDF2 [70].

However, further experiments are warranted to fully characterise the precise mechanisms by which these non-canonical m^6^A readers recognise m^6^A (direct recognition, m^6^A-induced RNA structural alteration, or interaction with other m^6^A readers [19,71]) and execute their functional actions in RNA stability.

## Multifaceted roles of the m^6^A pathway in LINE1 regulation

m^6^A appears on RNA from LINE1 (L1) elements, which are also transcribed by RNA polymerase II. The first study that identified m^6^A on L1 RNAs suggested a model wherein m^6^A promotes the degradation of L1 RNAs, exemplified by L1Md_F, through the YTHDC1–ZCCHC8/NEXT axis in mESCs [49]. A similar mechanism has also been observed in relation to other m^6^A-modified carRNAs in mESCs, but also worth noting that in this study *Mettl3* knockout mESC lines used were demonstrated to be hypomorphic, due to alternative splicing events bypassing the CRISPR-mediated indels [11]. Unlike PROMPTs and eRNAs, full-length L1 RNAs have a poly(A) tail. It remains in question how ZCCHC8/NEXT, which primarily targets non-polyadenylated nuclear RNA, is responsible for degrading L1 RNAs [49,72].

In contrast with this finding, a study conducted in K562 cells, a human immortalised myelogenous leukaemia cell line, observed a preference for m^6^A on intronic L1 RNAs. These intronic L1 RNAs are evolutionarily young and oriented towards the hosting gene. By comparing the steady-state L1 RNA levels with nascent L1 RNAs, this study suggested that m^6^A positively influences the expression of both autonomous L1 RNAs and co-transcribed L1 relics, and promotes L1 retrotransposition activity [73]. Moreover, these m^6^A-marked intronic LINE1 elements appear to act as ‘roadblocks' to RNA polymerase II within their host-long genes. In line with this positive correlation, another study utilising mESCs found that the majority of L1 RNAs are down-regulated when one of the components of m^6^A writer complex is constitutively knocked out (i.e. Mettl3, Mettl14, Wtap, Zc3h13). This led to the conclusion that m^6^A regulation on L1 RNAs operates through a post-transcriptional mechanism i.e. a stability-based control, as no differences were observed in histone modifications (H3K4me3, H3K27ac, and H3K9me3) at LINE1 loci [74].

As opposed to post-transcriptional regulation [75], another recent study reported that m^6^A can transcriptionally suppress LINE1 transcription in mESCs. They found that m^6^A transcriptionally represses retrotransposons via the YTHDC1–SETDB1–H3K9me3 pathway in mESCs with the observation that steady-state L1 RNAs are up-regulated upon conditional knockout (cKO) of *Ythdc1* or *Setdb1*, the enzyme that deposits H3K9me3 over LINE1 loci to repress their transcription. *Ythdc1* cKO induces the 2-cell (2C)-like transcriptional programme, largely through indirect regulation of *Dux*, *Zscan4*, and *MERVL*. However, this study raised another conflict: how does the loss of METTL3 or YTHDC1 could lead to both L1 RNAs and 2C marker gene *Dux* up-regulation? Specifically, as Percharde et al. [76] reported that L1 RNAs repress *Dux* transcription by recruiting Nucleolin/KAP1, thus resulting in a reciprocal expression relative to *Dux.* These conflicting results may stem from variations in the methods used to generate knockouts or potential secondary effects resulting from chronic knockouts, or the sensitivities of the technology to measure RNA half-life/stability. To provide clarity on the role of m^6^A in L1 RNA stability, employing an experimental system with acute depletion and/or complementation assays, together with time-resolved sensitive measurement, will be instrumental [22].

In addition to transcriptional and post-transcriptional roles of m^6^A on L1 RNA, another study proposed that m^6^A on L1 RNAs recognised by YTHDC1 regulates the formation of L1 RNA–Nucleolin in the nucleus, thus facilitating KAP1 recruitment on the 2C-related transcriptional programme in early mouse embryonic development [77]. Hwang et al. [78] using a reporter assay found that the m^6^A cluster in the L1 5′-UTR region serves as a docking site for eIF3 to enhance translational efficiency and promote the formation of L1 RNPs. Indeed, further investigations are required to elucidate how m^6^A alters the structure or scaffold of L1 RNAs and whether this structural change contributes to RNA stability. It remains an open question whether different m^6^A sites or clusters on L1 RNAs exert distinct roles, contributing to the observed functional diversity. This area of study holds great potential for advancing our understanding of m^6^A-mediated regulation in the context of LINE1 elements and early mammalian development.

## Conclusion and outlook

This review considers the current understanding in the field of how RNA modification m^6^A impacts on the regulation of RNA stability (Table 1). This layer of gene regulation is primarily orchestrated by m^6^A reader proteins along with their interacting partners. The reader-based recognition of m^6^A and its resultant impact on stability is influenced by developmental stages, cellular environments, and the RNA sequence/structure itself. The dual functions of m^6^A in RNA stability regulation underscore a fundamental question: are there any factors determining whether m^6^A recognition by different readers leads to RNA degradation versus stabilisation? Whilst it is established that YTHDFs and IGF2BPs contribute to m^6^A-dependent RNA degradation and stabilisation respectively, the precise features that dictate recognition by distinct readers of m^6^A sites or m^6^A-containing RNAs remain elusive. Exploring the phenomenon where distinct readers can bind to the same m^6^A moiety on RNAs raises interesting questions. It would be worthwhile to delve into the potential implications of binding competition as a mechanism for RNA stabilisation. This competition could function by preventing YTHDFs binding, offering a unique perspective on the complex interplay of different readers in regulating RNA stability. Furthermore, the relationship between the kinetics of stability and m^6^A stoichiometry remains to be clearly delineated. Considering the intricate regulation of RNA stability by various factors like microRNAs, RNA-binding proteins, poly(A) tails, and RNA structure, exploring how RNA modifications interact with these elements to govern stability demands deeper investigation.

**Table 1.** Summary for the role of m^6^A readers in RNA stability regulation

Role	Reader	m^6^A-containing RNA targets type	Cellular localisation	Species
RNA degradation	YTHDF1/2/3	mRNA, lncRNA, circular RNA	Cytoplasm	Human, Mouse, Drosophila, Zebrafish
	YTHDC1	carRNA, lncRNA, repeat RNA	Nucleus	Mouse
	FMR1	mRNA	Cytoplasm	Drosophila, Mouse
RNA stabilisation	IGF2BP1/2/3	mRNA, lncRNA	primarily in Cytoplasm, but also in Nucleus	Human, Mouse
	YTHDC1	mRNA (MYC)	Nucleus	Human, Mouse
	PRRC2A	mRNA (Olig2)	Cytoplasm	Mouse
	PRRC2B	mRNA (Sox2)	Cytoplasm	Mouse

Further exploration is also warranted to delve into the causal relationship between m^6^A and developmental or disease-related phenotypes, considering the broad impact of m^6^A on RNA metabolism that often results in substantial secondary effects when m^6^A pathways are chronically disrupted. Caution must be taken during the generation of *Mettl3* knockout, as alternative splicing events may bypass these deletions, potentially yielding a truncated METTL3 protein that retains catalytic activity [11]. These occurrences could underlie several conflicting findings. Acute depletion systems offer a solution by largely eliminating those unwanted secondary effects, thereby providing a clearer delineation of direct causality [22,74]. Additionally, the targeted editing of individual m^6^A modifications using RNA-guided RNA-targeting CRISPR systems [79–81], such as CRISPR–Cas13, presents another avenue to investigate the specific functions of individual m^6^A modifications without disturbing the entire m^6^A writing or erasing systems.

## Perspectives

- As one of the most prevalent internal RNA modifications, m^6^A influences various aspects of RNA metabolism, thereby regulating gene expression crucial to development and tissue functions. m^6^A dysregulation leads to diseases, including cancer.
- The modulation of RNA stability by m^6^A is largely driven by reader proteins that bridge to downstream molecular pathways, further influenced by RNA sequence context, developmental stage, and cellular environment.
- The intricate interplay between m^6^A and various RNA regulatory elements, including microRNAs, RNA-binding proteins, and RNA structure, forms a complex network that significantly influences RNA stability. A comprehensive understanding of these relationships is pivotal to fully elucidate the mechanisms governing RNA stability regulation.

## Open Access

Open access for this article was enabled by the participation of University of Oxford in an all-inclusive *Read & Publish* agreement with Portland Press and the Biochemical Society under a transformative agreement with JISC.

## Abbreviations

cKO: conditional knockout
co-IP: co-immunoprecipitation
KH: K homology
m^6^A: *N*^6^-methyladenosine
mESCs: mouse embryonic stem cells
PROMPT: promoter upstream transcript
PRRC2: proline-rich coiled-coil 2; nuclear exosome targeting complex (NEXT)
